# Peripheral immune characteristics and subset disorder in reproductive females with endometriosis

**DOI:** 10.3389/fimmu.2024.1431175

**Published:** 2024-11-28

**Authors:** Kai-Rong Lin, Pei-Xian Li, Xiao-hong Zhu, Xiao-fan Mao, Jia-Li Peng, Xiang-Ping Chen, Cui-Yao SiTu, Li-Fang Zhang, Wei Luo, Yu-Bin Han, Si-Fei Yu

**Affiliations:** ^1^ Institute of Translational Medicine, The First People'sHospital of Foshan, Foshan, Guangdong, China; ^2^ Department of Gynecology, The First People’s Hospital of Foshan, Foshan, Guangdong, China

**Keywords:** endometriosis, peripheral immune profile, immune cell subset, flow cytometry, diagnosis

## Abstract

Pathogenesis of endometriosis (EN) is still unknown, but growing evidence suggests that immune regulation may be important, and the pattern of peripheral immune changes in reproductive women with EN has yet to be fully explored. In this study, we conducted a comprehensive and systematic analysis of immune cell subsets within T cells, B cells, NK cells, and γδ T cells in peripheral blood (PB) samples from women with EN, women with uterine fibroids (UF) but without EN (UF-alone), and healthy controls using multi-parameter flow cytometry. Our findings revealed that UF, a common comorbidity of EN, exhibited similar peripheral immune features to EN, particularly in T cell and B cell immunity. Compared to healthy controls, we constructed the peripheral immune profile of EN. This profile highlighted that the immunopathogenic factors in EN predominantly relate to the immune disorder of B cells and their subsets, as well as the functional abnormalities within immune cell subsets of CD4^+^ T cells, CD8^+^ T cells, and γδ T cells. Moreover, using the random forest (RF) machine-learning method, we developed a diagnostic model that can effectively identify the patients with EN from healthy controls. The immune factors identified within this model could be pivotal for unraveling the immune pathogenic mechanisms of EN. Our study is the first to present a comprehensive depiction of the circulating immune features in EN, although the detailed roles and underlying mechanisms of these immune factors in the context of EN require further investigation.

## Introduction

Endometriosis (EN) is a common gynecological condition that affects a significant number of women globally. It is characterized by a growth of the active endometrial tissue, which normally lines the inside of the uterus, in places outside the uterine cavity, such as the ovaries, fallopian tubes, and the peritoneal lining of the pelvic cavity (1). The most common symptoms, including dysmenorrhea (painful periods), chronic pelvic pain, infertility, dyspareunia (painful intercourse), dyschezia (painful bowel movements), dysuria (painful urination), and menstrual irregularities, significantly impact a woman’s physical, emotional, and social well-being, leading to a reduced quality of life (2). It is estimated that approximately 10% of reproductive-age women are diagnosed with EN during their lifetime, about 50% to 80% of those with chronic pelvic pain, and up to 50% of those with infertility (1, 3). The true prevalence of EN in the general population could exceed current estimates, given that numerous cases may remain undiagnosed or misdiagnosed due to its non-specific symptoms, and the invasive nature of the diagnostic procedures required, such as diagnostic laparoscopy and histodiagnosis.

Treatment options for EN are aimed at managing symptoms and improving quality of life, including pain management, hormonal therapies, and in some cases, surgical intervention. However, there is currently no known cure for EN, and the disease tends to recur (4). EN is increasingly recognized as a systemic disease rather than one predominantly affecting the pelvis. It affects metabolism in the liver and adipose tissue, leads to systemic inflammation, and alters gene expression in the brain, resulting in increased pain perception and mood and anxiety disorders that are more common in women with EN (5, 6). The exact cause of EN is not fully understood, but it is believed to involve a combination of genetic, hormonal, immunologic, inflammatory, and environmental factors (7). The role of the immune system in EN has been suggested to play an important role in the pathogenesis of the disease. Women with EN are more likely to have other immune-mediated diseases, such as rheumatoid arthritis, systemic lupus erythematosus, autoimmune thyroid disorder, and inflammatory bowel disease, suggesting a shared pathophysiological basis and a systemic immune response (8). Considerable evidence suggests abnormal functioning of nearly all immune cell types in women with endometriosis. This is characterized by reduced T cell reactivity and NK cell cytotoxicity, polyclonal activation of B cells with heightened antibody production, elevated numbers and activation of peritoneal macrophages, as well as alterations in inflammatory mediators (9). Although several immunological abnormalities have already been reported, the role of the immune system in EN is not well established, particularly since the circulating immune landscape of EN is still lacking (10–12). Therefore, a better understanding of the activities of numerous cells involved in immune reactions in EN could lead to more effective treatments as well as noninvasive and convenient diagnoses for this challenging condition (13).

Taking into account the systemic nature of endometriosis, our study focused on identifying immune markers in peripheral blood. In this study, we aimed to provide comprehensive insights into the peripheral immune landscape in reproductive females with EN, including a total of 70 different immune cell subtypes of T cells, B cells, natural killer (NK) cells, gamma delta T (γδ T) cells, and their various functional subsets, which may contribute to clarifying the role of peripheral immune function in both initiation and progression of this disease. In addition, given that uterine fibroids (UF, also known as leiomyomata) involve similarly abnormal growth of uterine tissue and share symptoms such as abdominal pain, dyspareunia, and heavy menstrual bleeding with EN (14), often co-occurring in affected women and complicating the differential diagnosis and clinical management of EN (15), our study simultaneously included reproductive-aged women with UF (serving as non-EN disease control patients, UF-alone patients). Recent studies have suggested that both EN and UF exhibit overlap in their genetic etiology (16), and have also been associated with alterations in immune and endocrine systems that promote inflammation and abnormal hormonal environments linked to the development of chronic diseases (17). However, the immune similarities or differences between these two conditions remain underexplored. By using UF-alone patients as controls for EN patients (who were further subdivided into those with and without UF), we were able to gain significant insights into the immunological distinctions between EN and UF. This understanding may aid in elucidating the causal mechanisms of both conditions in the future.

## Materials and methods

### Study subjects

A total of 56 patients with EN, 35 UF-alone and 94 healthy donors participated in this study. The study protocol was approved by the Ethics Committee for Medical Research of the First People’s Hospital of Foshan, and all participants signed written informed consent before enrolling in the study. They were all females of productive age (ages 25-45), and there were no significant differences in age among groups (**Supplementary Figure 1**).

All patients recruited in this study underwent laparoscopic surgery for EN and/or UF. EN or UF in all cases were confirmed by histopathological examination. Peripheral blood (PB) samples from these patients were prospectively collected before laparoscopic surgery. They had never undergone any medication treatment within three months before laparoscopic surgery and had no history of malignancy, chronic inflammatory, or autoimmune diseases. The clinical characteristics of a total of 91 patients recruited in this study are shown in **Table 1** and **Supplementary Table 1**. There were no significant differences in BMI, menstrual cycle length, menstrual days of flow, phase of the menstrual cycle, and history of pregnancy between the EN and UF patients. The EN patients had the main comorbidities as UF (32/56, 57.14%), endometrial polyps (12/56, 21.43%), and mesosalpinx cysts (8/56, 14.29%). While in the UF-alone patient were with additional occurrences of endometrial polyps (10/35, 28.57%) and mesosalpinx cysts (2/35, 5.71%). Among the 56 women with EN, there were 35 (62.50%) women had ovarian endometriosis, 18 (32.14%) had adenomyosis and 51 (91.07%) had pelvic endometriosis. The severity of EN was determined according to the revised American Society for Reproductive Medicine (rASRM) classification (American Society for Reproductive Medicine, 1996). Excluding the 3 cases of pure adenomyosis, the EN patients were further categorized into those with early-stage disease (rASRM stages I-II, n=20) and those with advanced-stage disease (rASRM stages III-IV, n=33).

**Table 1:** Clinical characteristics of women with and without endometriosis.

Groups		EN (*n* = 56, 60.87%)	UF-alone (*n* = 35, 31.13%)	P Value
Age (years)^a^		38 (25-44)	37 (25-45)	0.718
BMI (kg/m^2^)^a^		22.19 (15.65-32.77)	22.99 (17.85-30.73)	0.178
Menstrual cycle length(days)^a^		29 (25-45)	30 (24-38)	0.138
Menstrual days of flow(days)^a^		6.25 (3-10)	6 (2.5-9.5)	0.460
**Menstrual phase (*n*)^b^**	Proliferative	39 (69.64%)	20 (55.56%)	0.134
	Secretory	16 (28.57%)	16 (44.44%)	
	unknown	1 (1.79%)	0	
**Comorbidities (*n*)**	Uterine fibroids	32 (57.14%)	35(100%)	/
	Endometrial polyps	12 (21.43%)	10 (28.57%)	0.486
	Mesosalpinx cyst	8 (14.29%)	2 (5.71%)	0.189
Maximum diameter of lesion location (mm)^1^		62 (8-116)	64 (6-142)	/
**Lesion site of uterine fibroids (*n*)^b^**	Intramural	30(93.75%)	33(94.29%)	0.570
	Subserosal	1(3.13%)	0	
	Intramural + Subserosal	0	1(2.86%)	
	unknown	1(3.13%)	1(2.86%)	
**Lesion site of endometriosis (*n*)**	Ovarian	35 (62.50%)	/	
	Uterine Myometrium	18 (32.14%)	/	
	Pelvic endometriosis	51 (91.07%)	/	
**Stages of endometriosis (r-ARMS,*n*)**	Stage I	18 (32.14%)	/	/
	Stage II	2 (3.57%)	/	
	Stage III	13 (23.21%)	/	
	Stage IV	20 (35.71%)	/	
	Not applicable (Adenomyosis)	3 (5.36%)	/	
**With symptoms (*n*)^b^**	Pain^2^	27 (48.21%)	4 (11.11%)	**0.0002**
	Abnormal uterine bleeding	15 (26.79%)	17 (47.22%)	**0.045**
	Infertility	3 (5.36%)	0	0.158
	Others^3^	17 (30.36%)	18 (50.00%)	0.058
**History of pregnancy (*n*)^b^**	yes	34 (60.71%)	26 (72.22%)	0.18
	no	17 (30.36%)	5 (13.89%)	
	unknown	5 (8.93%)	5 (13.89%)	
**Blood test^a,4^**	Serum CA125 (U/ml)	94.27 ± 76.73	18.60 ± 4.70	**0.00009**
	AMH (ng/ml)	3.09 ± 2.55	1.27 ± 0.53	**0.00002**
	WBC (×10^9^/L)	6.71 ± 1.74	6.36 ± 1.801	0.360
	RBC (×10^12^/L)	4.67 ± 0.46	4.71 ± 0.54	0.965
	Hb (g/L)	123.57 ± 16.29	122.86 ± 19.53	0.857
	Lym (×10^9^/L)	1.81 ± 0.49	1.93 ± 0.57	0.442
	Ne (×10^9^/L)	4.32 ± 1.58	3.89 ± 1.34	0.173
	NLR	2.56 ± 1.30	2.07 ± 0.68	0.113

a Median(range)/Mean ± SD, Mann-Whitney U test between the Endometriosis patients (EN) and Non-Endometriosis patients with uterine fibroids (UF-alone).

b Chi-square test between the two groups.

^1^The characteristics of the biggest lesion tissue for ectopic focus of EN patient or for UF-alone patients.

^2^Pain was defined as the presence of dysmenorrhea/dyschezia/dyspareunia/chronic pelvic pain.

^3^Others were referred as abnormal findings detected by ultrasound during a physical examination.

^4^WBC, white blood cells; RBC, red blood cells; Hb, hemoglobin; Lym, lymphocytes; Ne, neutrophils; NLR, neutrophils/lymphocytes rate. The Bold P-values indicate significant differences between groups.

Healthy controls in our study were selected through a comprehensive approach that included interviews, clinical questionnaires, and imaging exams. All healthy individuals who have been recruited voluntarily, self-report being in good physical condition, have shown normal results for all examination indicators in their health check-ups within one year, and have never been diagnosed with any disease. Their age and gender were matched with those of the disease.

### Sample preparation and multi-parametric flow cytometric analysis

Five milliliters of heparin-anticoagulanted peripheral blood were collected, and only the qualified samples such as being free from clots and hemolysis were advanced to the subsequent experiments. Samples were stored at 4°C and tested promptly within 48 hours.

**Figure 1:**
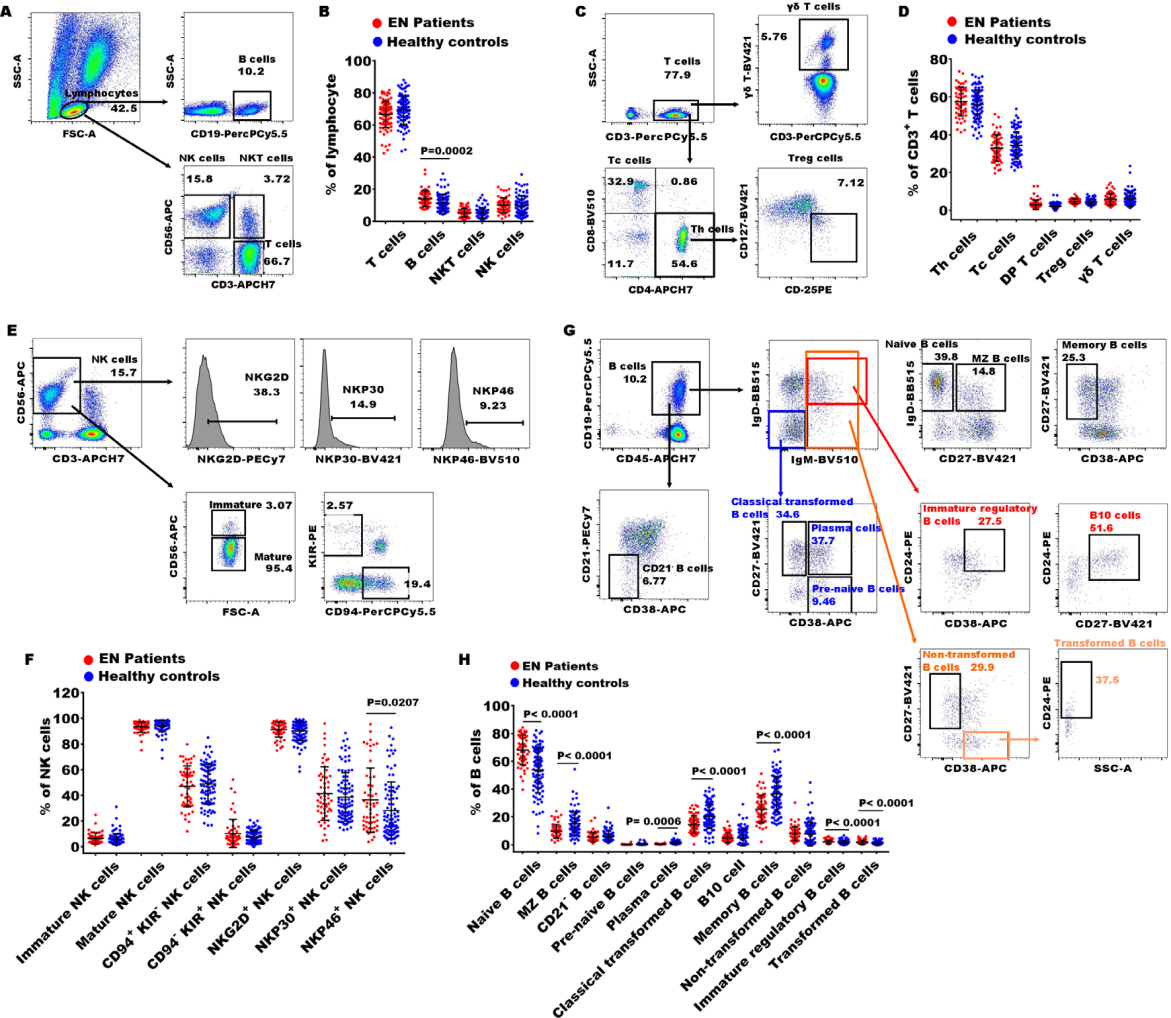
Gating strategies of flow cytometry and significances of immune indexes between patients with EN patients and healthy controls. **(A)** The representative dot diagrams showed the gating strategy of T cells, B cells, NKT cells, and NK cells; **(B)** statistical results indicated these subsets in the EN patients and the healthy controls. **(C)** The representative dot diagrams showed the gating strategy of different T cell subsets; **(D)** statistical results indicated these subsets in the EN patients and the healthy controls. **(E)** The representative dot diagrams showed the gating strategy of different NK cell subsets; **(F)** statistical results indicated these subsets in the EN patients and the healthy controls. **(G)** The representative dot diagrams showed the gating strategy of different B cell subsets; **(H)** statistical results indicated these subsets in the EN patients and the healthy controls.

**Figure 2:**
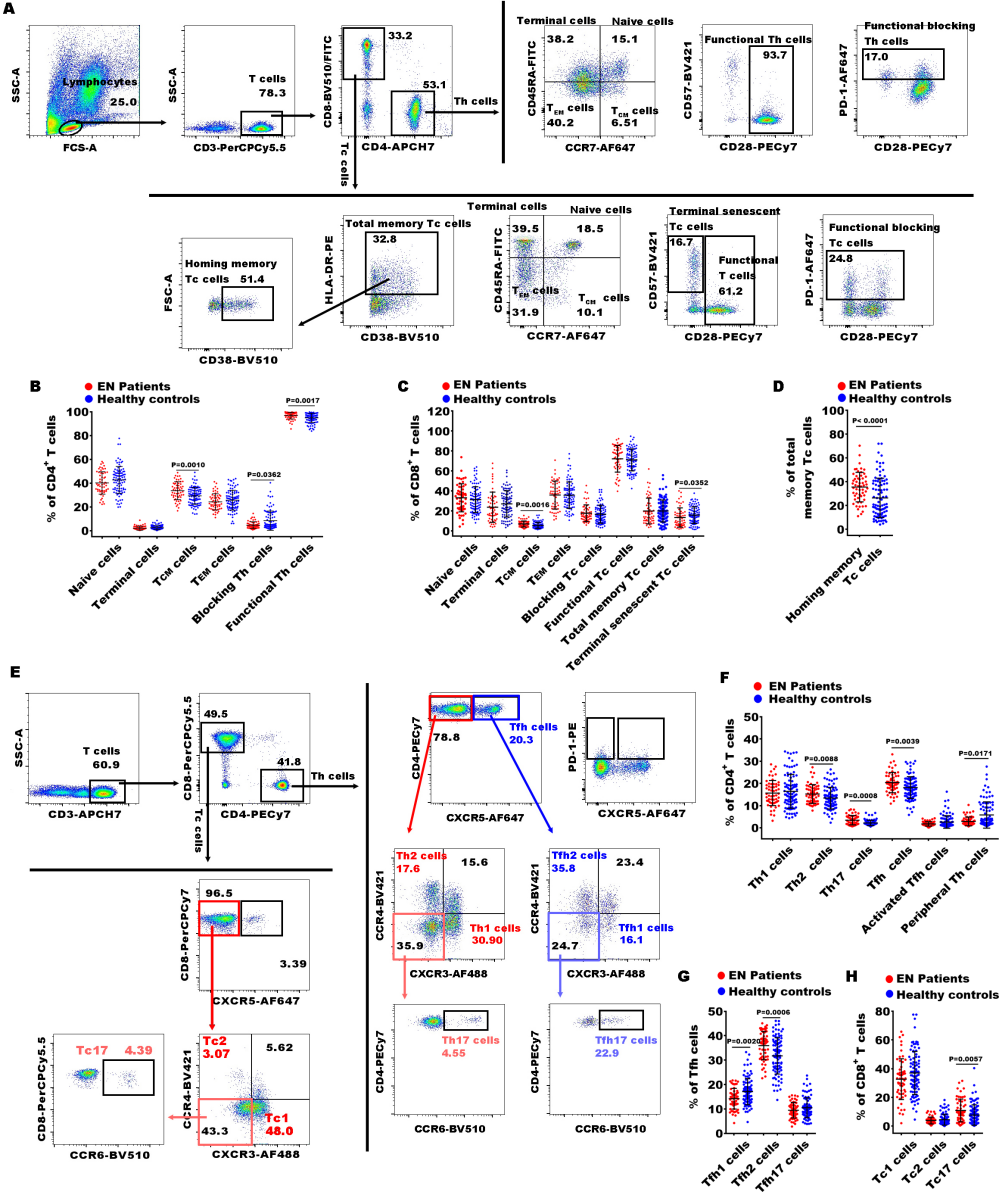
Gating strategies of flow cytometry and significances of CD4^+^ and CD8^+^ T cell subsets between EN patients and healthy controls. **(A)** The representative dot diagrams showed the gating strategy of different CD4^+^ and CD8^+^ T subsets representing functional state of the T cells; **(B–D)** comparison of the percentages of the above subsets of CD4^+^ and CD8^+^ T cells in the EN patients and the healthy controls. **(C)** The representative dot diagrams showed the gating strategy of different CD4^+^ and CD8^+^ T subsets representing distinct effector T subsets; **(F–H)** comparison of the percentages of the above subsets of the CD4^+^ and CD8^+^ T cell subsets in the EN patients and the healthy controls.

**Figure 3:**
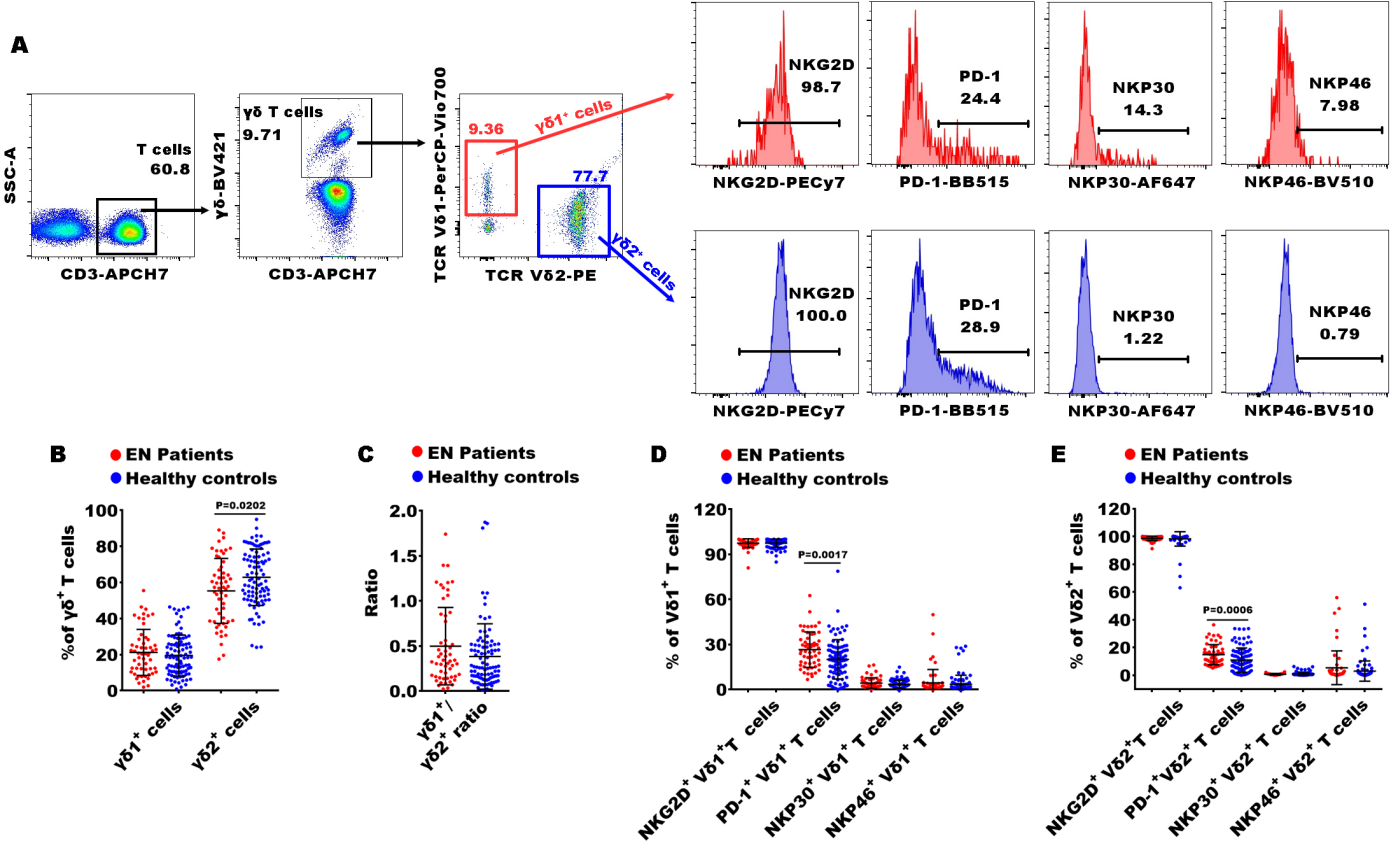
Gating strategies of flow cytometry and significances of **γδ** T cell subsets between patients with EN and healthy controls. **(A)** The representative dot diagrams and histograms showed the gating strategy of γδ T cells; **(B–E)** comparison of the percentages of γδ1^+^ and γδ2^+^ T cells, and the frequencies of NKG2D, PD-1, NKP30, and NKP46 in the EN patients and the healthy controls.

The antibody mixture was prepared specifically for the experiment, taking into account the characteristics of each antibody. The monoclonal antibodies were purchased from BD Bioscience and the relevant information was listed in **Supplementary Table 2**. Subsequently, 100 μl of venous blood and 10 μl of the prepared antibody mixture were added into flow cytometry tubes, which were gently agitated and incubated in the dark for 15 minutes at room temperature. After incubation, 1 ml of red blood cell lysis buffer (Solarbio, Beijing, China) was added into each tube, allowing the lysis to occur at room temperature for an additional 15 minutes and then centrifuged at 1000 rpm for 5 minutes. The cell pellets that formed were subsequently resuspended in 350 μl PBS, which were tested within 3 hours. BD LSRFortessa X-20 was used to detect the fluorescence, followed by data analysis using FlowJo software (version 10). A total of 70 various immune indexes were analyzed and the phenotypic and functional molecules identified for each subset were shown in **Supplementary Table 3**. The gating strategies for the analysis of each subset were depicted in **Supplementary Figure 2**, **Figures 1**–**3**. The percentages of 70 immune indexes from each patient and healthy donor were listed in **Supplementary Table 4**.

### Statistics

Statistical analysis was performed using IBM SPSS software (version 20.0, USA) and GraphPad Prism software (version 6.0, USA). The Mann–Whitney *U* test was used to determine the statistical difference between the two groups. The Chi-square test was used to analyze the frequency differences in categorical data. The relationship between the above immune indexes and clinical characteristics was performed using Spearman correlation analyses. All tests were two-sided, and significance levels were set to *P<0.05, **P<0.01, ***P<0.001, and ****P<0.0001. Unsupervised cluster analysis, principal component analysis (PCA), random forest (RF) machine-learning method, and the receiver operating characteristic (ROC) analysis were performed by R (version 3.4.3) and Python (version 3.8).

## Results

### EN and UF exhibit similar peripheral immune characteristics

EN and UF are two distinct gynecological conditions that involve the abnormal growth of uterine tissue. Recent studies have indicated a correlation between the two conditions, suggesting a common, partially genetic and endocrine etiology. This finding implies a heightened risk of comorbidity for individuals with EN who develop UF, and reciprocally for those with UF who experience EN (16, 17). To investigate the similarities and differences in the peripheral immune features between EN and UF, we subdivided the patients into distinct cohorts and conducted a comparative analysis of the 70 different peripheral immune indexes across these groups (**Figure 1A**). Overall, most of the 70 immune indexes were not significantly different between EN patients and UF-alone patients without EN, as well as between EN-alone patients without UF and UF-alone patients, EN patients with UF (EN + UF) and UF-alone patients, and the EN-alone and EN + UF patients.

**Figure 4:**
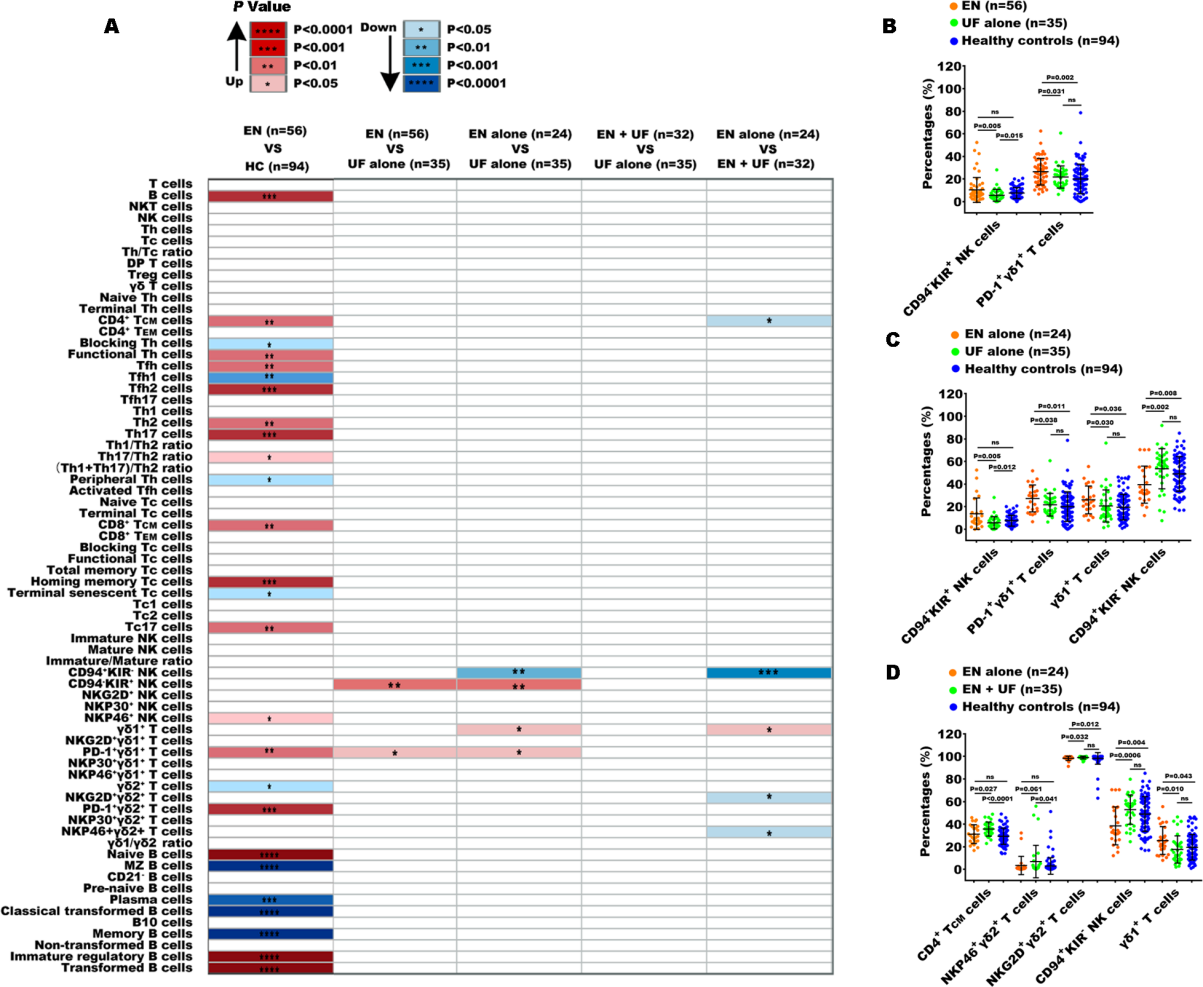
Peripheral immune indexes detected by multiparameter flow cytometry. **(A)** The overview of the significantly changed immune indexes between the patients with EN and the healthy controls (HC), and between the different groups from EN and non-EN patients. Up, the percentages of immune indexes were increased in the front group; down, the percentages of immune indexes were decreased in the front group. The significantly changed immune subsets among the EN patients, UF-alone patients, and HC **(B)**, among EN-alone patients, UF-alone patients, and HC **(C)**, among EN-alone patients, EN patients with UF (EN + UF) and HC **(D)**. *P<0.05, **P<0.01, ***P<0.001,****P<0.0001.

Comparing the 70 indexes between the EN and UF-alone groups, we found that only the percentages of CD94^-^KIR^+^NK cells and PD-1^+^γδ1^+^ T cells were increased in the EN group. Notably, PD-1^+^γδ1^+^ T cells exhibited a difference when compared to the healthy individuals (**Figures 4A, B**). To exclude the confounding effects of coexisting UF on the peripheral immune response in EN, we compared the 70 indexes between the EN-alone patients and the UF-alone patients. The results revealed that, besides the proportions of CD94^-^KIR^+^ NK cells and PD-1^+^γδ1^+^ T cells, which elevated in the EN-alone patients, there was an increase of the proportions of γδ1^+^ T cells and a decrease in the proportion of CD94^+^ KIR^-^ NK cells in this same EN-alone cohort (**Figure 4C**). Conversely, when comparing the EN with UF patients to the UF-alone patients, no significant differences were observed in any of the 70 immune markers (**Figure 4A**). We further compared the 70 indexes between the EN patients with and without UF, and the results showed that the percentage of γδ1^+^ T cells was increased in the EN-alone patients, while the percentages of central memory CD4^+^ T cells (CD4^+^ T_CM_), NKP46^+^γδ2^+^ T cells, NKG2D^+^γδ2^+^ T cells, and CD94^+^KIR^-^ NK cells were decreased in the EN-alone patients. Remarkably, only the percentages of γδ1^+^ T cells, NKG2D^+^γδ2^+^ T cells, and CD94^+^KIR^-^ NK cells showed significant differences between the EN-alone patients and the healthy cohorts (**Figure 4D**).

Taken together, the above findings demonstrate that EN and UF exhibit similar peripheral immune functions, particularly in T cell and B cell immunity, despite the detection of minor immune alteration in NK cell and γδ T cell subsets.

### Immune disorder of B cells and their subsets in PB from the patients with EN

To further elucidate the peripheral immune characteristics in patients with EN, we conducted a comparative analysis with the immune profiles of healthy subjects. First, we observed the percentages of T cells (CD3^+^), B cells (CD19^+^), NKT cells (CD3^+^CD56^+^), and NK cells (CD3^-^CD56^+^) in peripheral lymphocytes and found that only the percentage of B cells significantly increased in the EN patients compared to the healthy controls (**Figures 1A, B**). To further understand the alterations in immune function, we then examined the characteristics of T subsets, B subsets, and NK subsets. Within CD3^+^ T cells, the percentages of Th cells (helper T cell, CD4^+^), Tc cells (cytotoxic T cell, CD8^+^), DP T cells (double positive T cells, CD4^+^CD8^+)^, Treg cells (regulatory T cells, CD4^+^CD25^+^CD127^low/-^), and γδ T cells (γδ^+^) exhibited no significant alterations in the EN patients (**Figures 1C, D**).

Next, we also observed the changes in 8 immune indexes of NK cell subsets, including immature NK cells (CD3^-^CD56^+/bright^), mature NK cells (CD3^-^CD56^+/dim^), immature/mature NK cells ratio, CD94^+^KIR^-^ NK cells, CD94^-^KIR^+^ NK cells, NKG2D^+^ NK cells, NKP30^+^ NK cells, and NKP46^+^ NK cells. The results showed that only NKP46^+^ NK cells were significantly increased in the EN patients compared to the healthy individuals (**Figures 1E, F**). Notably, the percentage of NKP46^+^ NK cells was significantly elevated in the patients with advanced EN compared to those with early-stage EN.

Finally, the proportions of B cell functional subsets were analyzed. We observed that the proportions of naive B cells (CD27^-^IgD^+^), immature regulatory B cells (IgD^+^IgM^+^CD24^+^CD38^+^), and transformed B cells (CD27^-^CD38^+^IgM^+^CD24^+^) were obviously increased in the EN patients compared to the healthy controls, and MZ B cells (Marginal Zone B cells, CD27^+^IgD^+^), plasma cell (IgD^-^IgM^-^CD27^+^CD38^+^), classical transformed B cells (IgD^-^IgM^-^CD27^+^CD38^-^), and memory B cells (CD27^+^ CD38^-)^ were remarkably decreased (**Figures 1G, H**). These findings indicated that the disorder of B cell subsets might play important roles in pathophysiology of EN.

### Abnormalities within immune cell subsets of Th cells, Tc cells, and γδ T cells in PB from the patients with EN

Despite the absence of significant alterations in the levels of CD3^+^ T cells, Th cells, Tc cells, and γδ T cells in patients with EN, current evidence suggests that T cells play an important role in pathogenesis of EN through multiple mechanisms, including immune dysregulation, altered cytokine production, promotion of angiogenesis, and interactions with other immune cells (18). Consequently, it is essential to conduct a more detailed analysis of the T cell subsets in the PB of patients with EN to uncover any potential imbalances that may be present. Here, we analyzed the significant alterations in 15 immune indexes of Th cell subsets, 12 of Tc cell subsets, and 11 of γδ T cell subsets in patients with EN compared to the healthy controls.

In Th cell subsets, we observed that the percentages of central memory CD4^+^ T cells (CD4^+^ T_CM_ cells, CD4^+^CD45RA^-^CCR7^+^), functional Th cells (CD4^+^CD28^+^ T cells), Th2 (CD4^+^CXCR5^-^CXCR3^-^CCR4^+^), Th17 (CD4^+^CXCR5^-^CXCR3^-^CCR4^-^ CCR6^+^), Th17/Th2 ratio, Tfh (T follicular helper cells, CD4^+^CXCR5^+^ T cells), and Tfh2 (CD4^+^CXCR5^+^CXCR3^-^CCR4^+^) were significantly increased in EN patients compared to healthy controls, while the levels of functional blocking CD4^+^ T cells (blocking Th cells, CD4^+^PD-1^+^), Tfh1 (CD4^+^CXCR5^+^CXCR3^+^CCR4^-^), and peripheral helper T cells (peripheral Th cells, CD4^+^CXCR5^-^PD-1^+^) were markedly reduced (**Figures 2A, B, E–G**). Within the Tc cell subsets, the percentages of central memory CD8^+^ T_CM_ cells (CD8^+^ T_CM_ cells, CD8^+^CCR7^+^CD45RA^-^), homing memory Tc cells (CD8^+^HLADR^+^CD38^+^), and Tc17 cells (CD8^+^CXCR5^-^CXCR3^-^CCR4^-^CCR6^+^) showed significant increases in the EN patients compared to the healthy controls, while the percentage of terminal senescent Tc cell (terminal senescent Tc cells, CD8^+^CD28^-^CD57^+^) exhibited a significant decrease (**Figures 2A, C-E, H**). Within the γδ T cell subsets, the percentages of inhibitory γδ1^+^ T cells (PD-1^+^γδ1^+^) and inhibitory γδ2^+^ T cells (PD1^+^γδ2^+^) were significantly elevated in the EN patients compared to the healthy controls, and the percentage of γδ2^+^ T cells was significantly reduced (**Figure 3**).

### Potential diagnostics value of peripheral immune indexes for EN

The above findings indicate that the specific immune indexes we examined can reflect the peripheral immunological alterations in EN. Therefore, we conducted a further analysis to assess the potential diagnostic efficiency of these immune indexes for EN, with the aim of establishing a non-invasive method for early detection and monitoring of disease progression.

Unsupervised clustering analysis and principal component analysis (PCA) were employed to preliminarily assess the effectiveness of distinguishing the EN patients from the healthy controls based on both the total 70 immune indexes and the 25 immune indexes that identified significant differences. The results from the two assessment methods were in general consistent with each other. However, the effectiveness was insufficient for differentiating the EN patients from the healthy controls based on neither the 70 immune indexes (**Supplementary Figures 3A, B**) nor the 25 specific immune indexes (**Supplementary Figures 3C, D**). Nonetheless, the composition of the 25 specific immune indexes showed a relatively better ability to differentiate the two groups.

**Figure 5:**
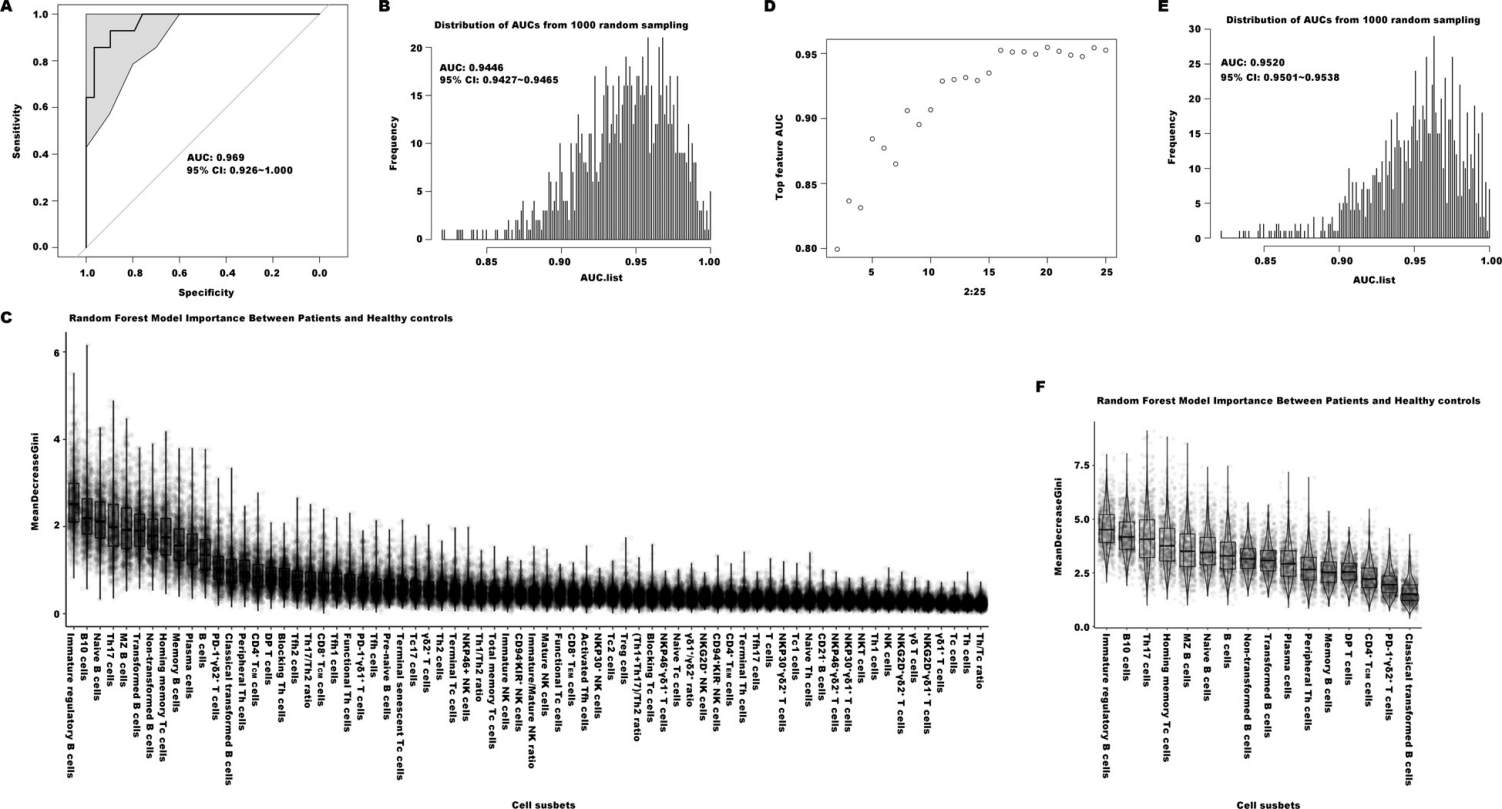
The diagnostic models of distinguishing the patients with EN from healthy controls. **(A)** The ROC curve of the efficiency of the diagnostic model constructed by one of random samplings using 70 immune indexes. **(B)** The AUC distribution of ROC curves from 1000 random samplings of the efficiency of the diagnostic model with 70 immune features. **(C)** The importance ranking calculated by the random forest model of the 70 immune cell subsets. **(D)** The average AUC distribution of ROC curves of the efficiencies of diagnostic models constructed by top important immune features. **(E)** The AUC distribution of ROC curves from 1000 random samplings of the efficiency of the diagnostic model with top 16 important immune features. **(F)** The importance ranking calculated by the random forest model for the top 16 important immune features.

The Random Forest (RF) machine learning technique was further used to establish an immunological diagnostic model for EN patients. The RF model could infer the significance of the proportion of each immune cell subset through a combination of classification and regression analyses. We designated 75% of individuals from both the EN patients and the healthy donors as the training set, and the remaining individuals were set aside as the test set. We conducted 1000 times of random sampling and used the averaged results to reduce potential variability. The diagnostic ability was assessed by the area under the curve (AUC) metric, derived from receiver operating characteristic (ROC) analysis. The model constructed using the total 70 immune indexes generated one ROC curve with an AUC value of 0.969 (95% CI = 0.926-1.000, as depicted in **Figure 5A**). The distribution of AUC values from 1000 random samplings was presented in **Figure 5B**, with an average AUC value of 0.945 (95% CI = 0.943-0.947). The importance ranking of each of the 70 indexes calculated by the RF model was displayed in **Figure 5C**. To establish an effective and convenient diagnostic model, we identified the essential minimum feature set from the 70 immune indexes by evaluating their respective ROC rates. As seen in **Figure 5D**, the AUC values of these models became stabilized when utilizing the top 16 most important indexes (including Immature regulatory B cells, B10 cells, Th17, and others as listed in **Figure 5F**, which were ranked by their importance). Its diagnostic ability was evaluated by the same approach, with an average AUC value of 0.952 (95% CI = 0.950-0.954, **Figure 5E**).

Despite there were some overlap in peripheral immune profiles between EN and UF patients, we were still interested in exploring the diagnostic performance of a model distinguishing between EN and UF using the RF method. Therefore we tried to construct an immunological diagnostic RF model based on the 70 indexes for identifying EN patients from UF. The outcomes showed that the average AUC value of the model from 1000 random samplings was also very low, only 0.582 (95% CI = 0.577-0.587, shown as **Supplementary Figures 4A–C**), and the model with the 8 most impactful indexes was the most simple and practical, with an average AUC value of 0.745 (95% CI = 0.7391-0.751, shown as **Supplementary Figures 4D–F**). The top 8 most impactful indexes included in this model respectively were CD94^-^KIR^+^ NK cells, Tfh17/Th2, plasma cells, NKT cells, immature regulatory B cells, PD1^+^γδ1^+^ T cells, CD94^+^KIR^-^ NK cells, and NKP30^+^ NK cells.

### Correlation between peripheral immune indexes and clinical characteristics

**Figure 6:**
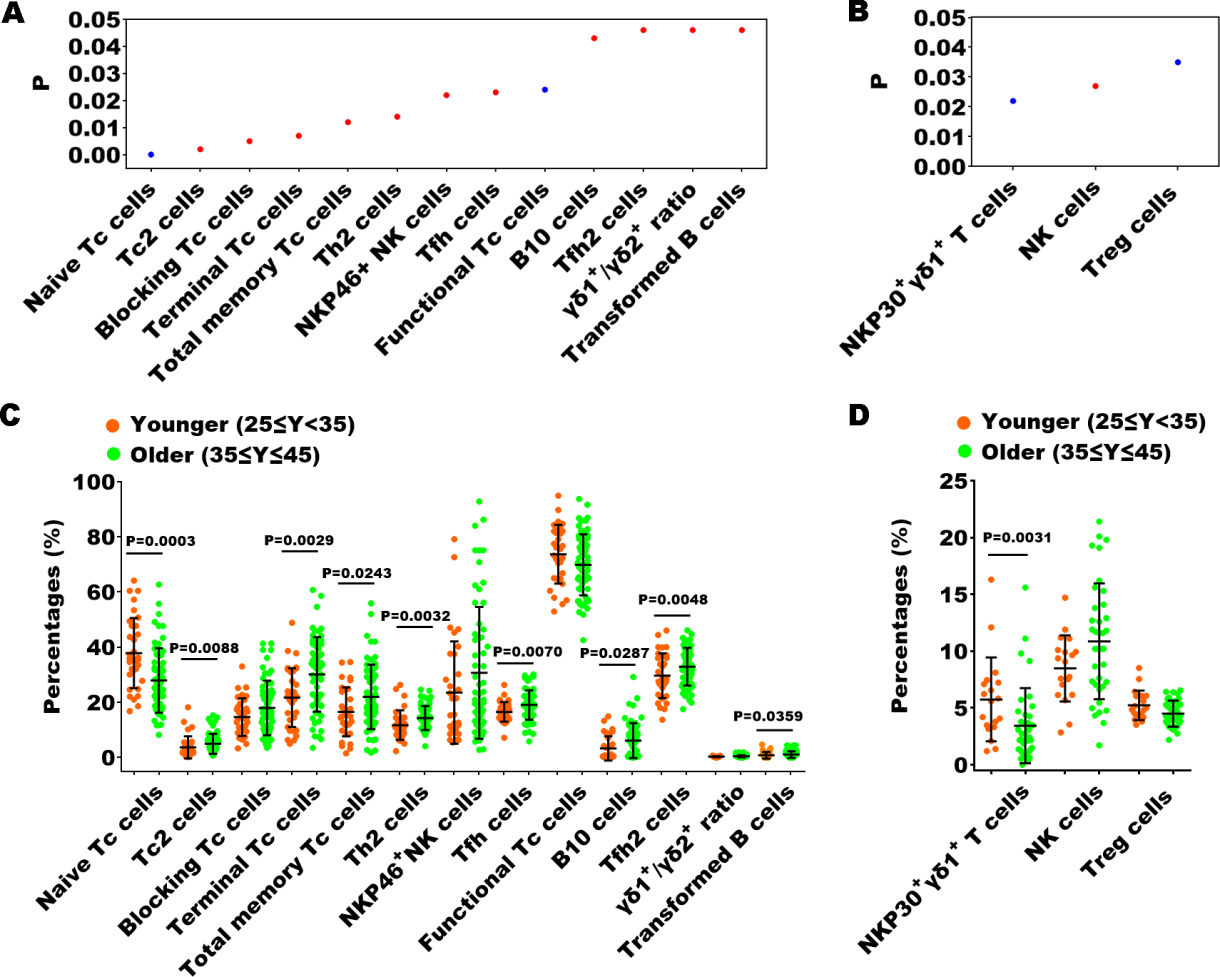
The relationship between the percentages of immune indexes and chronological age of healthy controls or the patients with EN. **(A, B)** The immune indexes that with significant correlations between their percentages and chronological age in healthy cohorts and in patients with EN. *P* < 0.05, Spearman correlation analysis; red, positive correlation; blue, negative correlation. **(C, D)** Comparison of the percentages of the immune indexes that with significant correlations between the younger and older individuals in the healthy cohorts and in the patients with EN, Mann-Whitney U tests.

Previous studies have confirmed that peripheral immunity is influenced by advancing chronological age (19, 20). To investigate the possible impact of chronological age on peripheral immune indexes, we employed Spearman correlation analysis to identify which specific immune indexes exhibited a statistically significant correlation with age. In the healthy cohort, 13 out of 70 immune indexes showed a significant correlation with age (**Figure 6A**), whereas in the EN patient cohort, only 3 immune indexes exhibited a significant correlation with age (**Figure 6B**). Subsequently, we stratified participants into younger (ages 25-35) and older (ages 35-45) subgroups, and performed Mann-Whitney U tests to assess differences in the immune indexes between the two subgroups, thereby further substantiating the influence of age on these indicators. We found that within the healthy cohort, there were still 9 immune indexes with significant differences between the two age groups among 13 immune indexes (**Figure 6C**). Notably, naive Tc cells exhibited an inverse relationship with age, whereas the remaining eight parameters demonstrated a direct correlation with increasing age. Within the EN patient cohort, a significant age-related difference was observed for only one immune indicator, the proportion of NKP30^+^ γδ1^+^ T cells, which inversely correlated with age (**Figure 6D**). This suggested that age was not a main factor affecting immune function in the context of EN disease.

**Figure 7:**
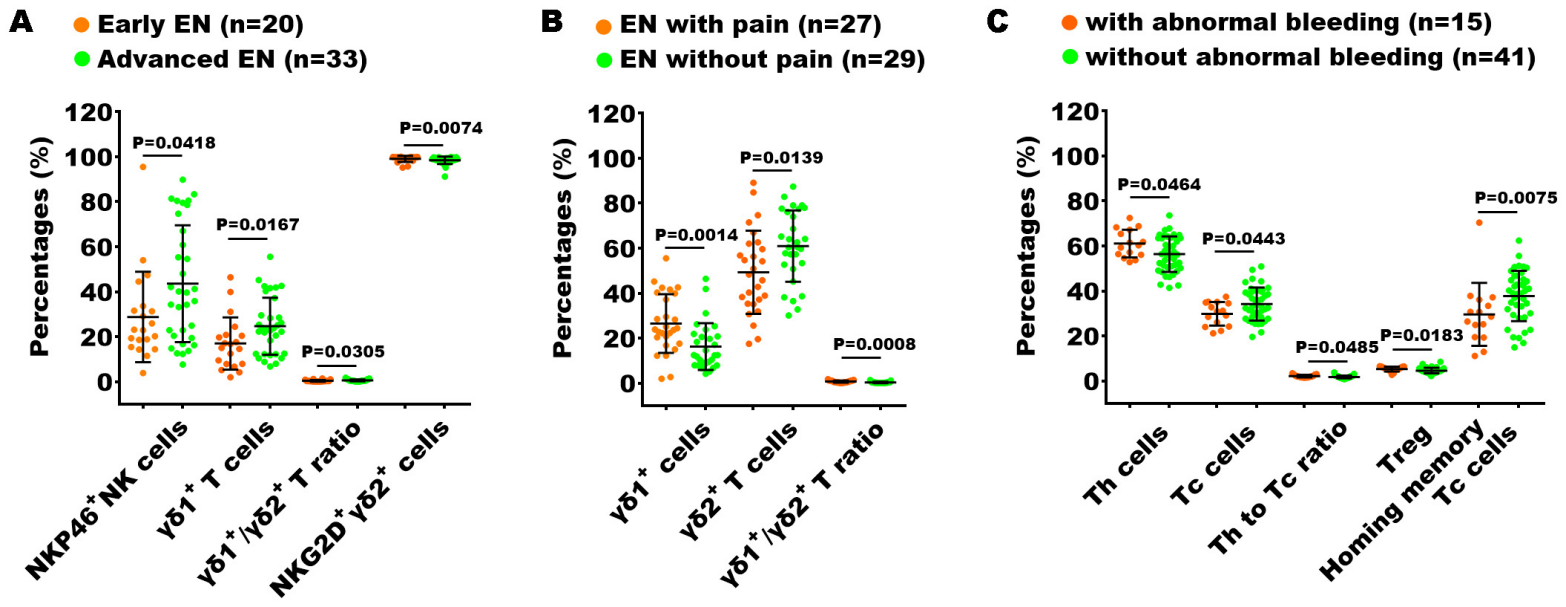
Analysis of the relationship between peripheral immunity in EN patients and their clinical stage and symptoms. The significantly changed immune indexes between EN patients with I + II stages (Early EN) and III + IV stages (Advanced EN) **(A)**, between EN patients with and without pain symptoms **(B)**, and between EN patients with and without abnormal uterine bleeding **(C)**.

Furthermore, we also explored the relationship between immune dysfunction and both disease severity and the presence or absence of clinical symptoms (including pain and abnormal uterine bleeding) in patients with EN. In the analysis of the 70 indexes across the early-stage and advanced-stage EN patients, we found that the percentages of NKP46^+^ NK cells, γδ1^+^ T cells, and the ratio of γδ1^+^ T cells to γδ2^+^ T cells (γδ1/γδ2 T ratio) in the advance EN patients were higher than in the early EN patients, while the percentage of NKG2D^+^ γδ2^+^ T cells were notably reduced (**Figure 7A**). We conducted the same analysis across patients with pain and those without pain, as well as across patients with abnormal uterine bleeding and those without. The results showed that the percentage of γδ1^+^ T cells and the γδ1/γδ2 T cell ratio were significantly higher in patients with pain symptoms compared to those without pain, while the percentage of γδ2^+^ T cells exhibited the opposite trend (**Figure 7B**). Additionally, in patients with abnormal uterine bleeding, the percentage of Th cells, the Th/Tc ratio, and the percentage of Treg cells were significantly elevated, whereas the percentage of Tc cells and homing memory Tc cells showed a significant decrease compared to those without abnormal uterine bleeding (**Figure 7C**).

Additionally, we performed Spearman correlation analysis to understand the relationship between the percentages of the peripheral immune indexes and the clinical characteristics of the EN patients, including BMI, menstrual cycle length, menstrual days of flow, maximum diameter of lesion location, as well as the laboratory test results of cancer antigen 125 (CA125), anti-Müllerian hormone (AMH), white blood cells (WBC), red blood cells (RBC), hemoglobin (Hb), lymphocytes (Lym), neutrophils (Ne) and the ratio of neutrophils/lymphocytes (NLR). The immune indexes that exhibited significant correlations with clinical features are listed in **Table 2**. Although the potential reasons for these correlations are not yet clear, these results can provide some additional insights for other research on the disease.

**Table 2:** The relationship between immune indexes and clinical phenotypes in the patients with EN.

Clinical phenotype	Immune index	Correlation coefficient (*r*)	*P* Value
BMI (*n*=55)	B cells	0.359**	0.007
	NKP30+ NK cells	-0.305*	0.022
	NKP46+ NK cells	-0.381**	0.004
	NKG2D+γδ2+ T cells	0.273*	0.042
	Naive B cells	0.345**	0.009
	Classical transformed B cells	-0.281*	0.036
	Memory B cells	-0.301*	0.024
	Non-transformed B cells	-0.272*	0.042
Menstrual cycle length (*n*=56)	Blocking Th cells	-0.330*	0.013
	Peripheral Th cells	-0.297*	0.026
	Activated Tfh cells	-0.323*	0.015
	NKP46+ NK cells	0.345**	0.009
Menstrual days of flow (*n*=56)	Terminal Th cells	-0.314*	0.019
	Th17 cells	0.336*	0.011
	Th17/Th2 ratio	0.323*	0.015
Maximum diameter of lesion location (*n*=38)	γδ1+ T cells	0.358**	0.007
	NKG2D+γδ2+ T cells	-0.355**	0.007
	γδ1/γδ2 ratio	0.324*	0.015
WBC (*n*=56)	Treg cells	-0.327*	0.014
	Tc2 cells	-0.288*	0.031
	NKG2D+γδ1+ T cells	-0.285*	0.033
	MZ B cells	-0.276*	0.039
RBC (*n*=56)	Double-positive T cells	-0.338*	0.011
	(Th1+Th17)/Th2 ratio	0.270*	0.044
	NKG2D+γδ1+ T cells	-.312*	0.019
Hb (*n*=56)	Th cells	-.299*	0.025
	Tc cells	0.363**	0.006
	Th to Tc ratio	-0.356**	0.007
	Double-positive T cells	-0.278*	0.038
	Treg cells	-0.308*	0.021
	CD4+ TEM cells	-0.274*	0.041
	Blocking Th cells	0.301*	0.024
	Peripheral Th cells	0.305*	0.022
	Activated Tfh cells	0.326*	0.014
	Tc17 cells	0.268*	0.046
Lym (*n*=56)	Tfh cells	-0.382**	0.004
	NKP46+ NK cells	-0.345**	0.009
	MZ B cells	-0.265*	0.048
Ne (*n*=56)	Treg cells	-0.291*	0.029
	NKG2D+γδ1+ T cells	-0.311*	0.02
CA125 (*n*=32)	γδ1+ T cells	0.350**	0.009
	γδ1/γδ2 ratio	0.340*	0.011
AMH (n=26)	Activated Tfh cells	-0.309*	0.021
	NKG2D+γδ1+ T cells	-0.275*	0.04
	NKG2D+γδ2+ T cells	-0.350**	0.008

Data were presentd as correlation coefficient (r) and P value of Spearman correlation analyses; *P<0.05, **P<0.01, No sinificant correlation were indentified between the percentages of any immune indexes and NLR (neutrophils/lymphocytes rate) of patients; WBC, white blood cellss; RBC, Red blood cellss; Hb, hemoglobin; Lym, lymphocytes; Ne,neutrophils; CA125, cancer antigen 125; AMH, anti-Müllerian hormone.

Data were presented as correlation coefficient (r) and P value of Spearman correlation analyses; *P<0.05, **P<0.01, No significant correlation were identified between the percentages of any immune indexes and NLR (neutrophils/lymphocytes rate) of patients; WBC, white blood cells; RBC, Red blood cells; Hb, hemoglobin; Lym, lymphocytes; Ne,neutrophils; CA125, cancer antigen 125; AMH, anti-Müllerian hormone.

## Discussion

EN and UF are the two most common gynecological diseases, data arising from various large-scale observational studies in the last decade show a relatively high comorbidity incidence of 57.9% to 87.1% in UF patients with EN (21–23). In our study, we also found a similarly high comorbidity rate of 57.14% in patients with EN who also have UF. The comorbidity between the two conditions is a significant medical concern and needs to be taken into account when treating either EN or UF. It was important to clarify whether EN and UF arose through a common mechanism, or whether one subsequently led to the development of the other. Recent evidence suggests that these two conditions share some common genetic foundations and are both influenced by estrogen-related pathophysiology (15); however, comparative research on their immune characteristics is lacking. In the current study, we first and systematically investigate the peripheral immune characteristics in reproductive females with EN and/or UF.

We examined a detailed comparison of αβ T cell, γδ T cell, NK cell, and B cell subsets in both the EN and the non-EN patients, with or without UF. Our findings revealed that EN and UF have similar peripheral immune features, particularly in αβ T and B cell immunity. Only a few subsets of NK and γδ T cells, marked by inhibitor receptors (such as CD94, KIR/NKB1, and PD-1), activating receptors, and natural cytotoxicity receptors (such as NKP46, NKP30, and NKG2D), showed slight differences between them. Interestingly, γδ1^+^ T cells exhibited a heightened state of activation or even signs of exhaustion in PB of EN, as evidenced by elevated levels of PD-1 expression. Furthermore, an increased number of γδ1^+^ T cells was observed in the advanced EN patients. Besides, γδ2^+^ T cells in PB of EN demonstrated reduced activation and cytotoxic capabilities, which was reflected by the decreased expression of NKG2D. In contrast to UF, our data suggested that the pathogenesis of EN might be associated with dysregulated immune responses of γδ1^+^ T cells and γδ2^+^ T cells. Moreover, compared to the UF-alone group, NK cells with lower expression of inhibitory receptor CD94 and higher expression of activating and cytotoxicity receptor NKP46 in PB of EN displayed enhanced cytotoxic activity. Interestingly, we observed that the peripheral immune profiles of the EN + UF group were more similar to those of the UF-alone group than to those of the EN-alone group. This observation might indicate that the peripheral immune system of patients with EN is more vulnerable to the effects of comorbid conditions, a consideration that should be taken into account when devising treatment strategies for EN, particularly about immunotherapeutic interventions.

In this study, we primarily focused on the peripheral immune features in EN patients. Based on the comparison with healthy controls, we found immune disorder within B cell subsets in the EN patients. B cells are known as lymphocytes that play a key role in the humoral immune response by producing antibodies that target and neutralize various antigens. A previous review (24) evaluated 22 studies that focused on the roles of B cells in EN, and almost all of them reported an increased number of B cells, along with their increased activation and/or production of autoantibodies. However, the mechanisms underlying B cells and autoantibodies in the development of this disease are still not well understood. Investigation into the various subsets of B cells in PB of EN could provide insights into the abnormal patterns of B cell differentiation and activation. Consistent with the previous reports, our research revealed an increase in B cells, naive B cells, immature regulatory B cells, and transformed B cells.

In addition, decreases in MZ B cells, plasma cells, classical transformed B cells, and memory B cells were observed in the EN compared with healthy donors. MZ B cells, a subset of innate-like B cells, produce broad-spectrum antibodies and participate in quick responses to blood-borne pathogens and secretion of natural antibodies that help to clear apoptotic cell debris (25). Plasma cells, also known as effector B cells, are primarily responsible for the production and secretion of antibodies to combat pathogens (26). They are derived from activated B cells and have undergone a process called class-switch recombination and somatic hypermutation in the germinal center, which enhanced the affinity of their antibodies. Classical transformed B cells represent one of the subsets that complete the transformation in this process (27). Memory B cells, although not directly part of the effector B cell populations, are important for long-term immunity (28). They are a subset of B cells that have previously encountered an antigen and can quickly differentiate into plasma cells upon re-exposure to the same antigen, thereby mounting a rapid and robust immune response. All of these B cell subsets decreased in PB of EN suggests the attenuation of B cell effector function might be involved in the development of EN.

Furthermore, it has become increasingly recognized recently that B cells can have a substantial influence on immune system through various mechanisms that are independent of their traditional antibody-mediated functions (29). For instance, MZ B cells have been observed to emulate the antigen-presenting capabilities of conventional dendritic cells (cDCs), thereby engaging in the presentation of antigens to CD4^+^ T cells, which in turn regulates their immune reactions (30). Consequently, it is essential to enhance our comprehension of the mechanisms of the B cell immune-modulating effect in addition to antibody production (29, 31). This understanding may be important for the pathogenic mechanisms of EN but has not yet been fully investigated.

T cells are the major cell constituents of adaptive immune system. Many previous studies have investigated the roles of Th and Tc cells in the pathophysiology of EN. Data from most of these studies focusing on the general Th and Tc cells in PB indicate no difference between patients with EN and controls (32, 33), which is consistent with our results. Further investigating the functional abnormalities and the distribution of subsets within Th and Tc cells is needed. Here, we assessed several main subsets identified with a distinct expression of surface molecules and chemokines. A previous study reported a higher level of CD4^+^ T_CM_ cells and CD8^+^ T_CM_ cells in PB of EN compared to healthy controls (34); our findings also revealed an increase in both CD4^+^ and CD8^+^ T_CM_ cells, along with elevated levels of homing memory Tc cells in the PB of EN, which might represent the distinctive immune imprint of EN on peripheral memory T cells. These observations indicate enhanced immune surveillance and a readiness to respond to potential antigenic challenges. The significant increase in homing memory Tc cells, which are specialized for targeted migration to specific tissues or sites of inflammation (35), further highlights the potential for localized immune responses in EN. Besides, we also observed a higher percentage of functional Th cells and lower percentages of blocking Th cells and terminal senescent Tc cells, suggesting less immunosuppression in PB of EN. These could be indicative of the immune system’s adaptive response to maintain an alert stance in the face of persistent or intermittent inflammatory processes in EN (36). Notably, despite no significant difference in the percentages of Th cells, Tc cells and Treg cells in PB between EN patients and healthy controls, we observed a notable distinction among EN patients with and without abnormal uterine bleeding. Specifically, patients with abnormal uterine bleeding exhibited higher expression levels of Th cells and Treg cells compared to those without abnormal uterine bleeding, whereas the opposite trend was observed for Tc cells and homing memory Tc cell. This suggests that the imbalance of Th cells (especially Treg cells) and Tc cells (especially homing Tc cells) may contribute to abnormal uterine bleeding, which warrants further in-depth investigation. On the other hand, upon TCR stimulation, naive T cells can be differentiated into distinct effector T cell subsets under different cytokines and costimulatory stimulation, such as Th1, Th2, Th17, and Tfh for CD4^+^ T cells, as well as Tc1, Tc2, and Tc17 cells for CD8^+^ T cells, which are defined by indicated phenotypes and exert distinct functions in host defenses (37, 38). In our dataset, we observed that an increase of Th2, Th17, Th17/Th2, Tfh, Tfh2, and Tc17 cells, and a decrease of Tfh1 and peripheral Th cells in the PB of EN.

Most studies support the idea that Th1 and Th2 balance maintains physiological homeostasis and once the balance is disturbed, various diseases such as allergies and autoimmune diseases will emerge (39). Consistent with our findings, it has been reported that a shift towards Th2 immune response with increasing type 2 cytokines detected in the peritoneal fluids and serum of EN patients, may account for the disease progression (40). Although the role of Th1/Th2 immune response bias in the pathogenesis of EN is not systematically understood, targeting this imbalance to eliminate the immune tolerance and recover normal immune regulation holds promise as a potential therapeutic strategy for EN (39).

Th17 and Tc17 cells are likely involved in several chronic and autoimmune inflammatory diseases (41). Peripheral blood Th17 and Tc17 cells play an important role in the pathogenesis of psoriasis, and the clinical improvement is correlated with a reduction in the inflammatory response mediated by Th17 and Tc17 cells in the PB of patients (42). Various studies have confirmed that both IL-17 and Th17 cells are linked to EN. Their expression is up-regulated in serum, peritoneal fluids (PF), and endometriosis lesions from EN patients (43). Th17-related effector cytokine IL-17 can initiate an inflammatory response through recruiting, activating, and driving the migration of neutrophils and M2 macrophages (44, 45), and further leads to the proliferation, growth, and invasion of ectopic foci, promotes the immune escape of ectopic foci and the progression of EN (43). Our data showing an increase in Th17 and Tc17 cells is consistent with these previous reports, and it might suggest the potential therapeutic value of targeting the Th17 and Tc17 cells in the treatment of EN.

Tfh cells are involved in humoral immune responses by promoting B cell proliferation, maturation, activation, and antigen-specific antibody production (46). They have also been identified as distinct subsets of effector functions: Tfh1, Tfh2, and Tfh17 cells. Tfh1 cells produce interferon-γ (IFN-γ), Tfh2 cells produce IL-4 and IL-13, Tfh17 cells produce IL-17, and all these subsets produce IL-21. It is acknowledged that only Tfh2 and Tfh17 cells could efficiently induce naive B cells to produce immunoglobulins via IL-21, while Tfh1 cells could not help B cells (47). The imbalance of Tfh subsets plays a key role in the pathogenesis of various autoimmune diseases. Although the patterns of alternation in Tfh subsets differed among the diseases, many researchers have identified Tfh2 and Tfh17 cells as pathogenetic subsets (48). To the best of our knowledge, the present findings are the first to reveal an increase in Tfh cells, mainly Tfh2 cells in the PB of EN, alongside a reduction in Tfh1 cells. Further research regarding the role of Tfh subsets in EN are of particular importance, which may enlighten us to more suitable therapeutic options for the disease.

NK cells and γδ T cells provide the first line of defense against virus-infected cells and tumors, with their activity being modulated by the equilibrium between inhibitory and activating receptors. Within NK cells, only the frequency of NKP46^+^ NK subsets were significantly higher in the PB of EN compared to healthy controls, indicating the enhanced cytotoxic activity in peripheral NK cells. Previous studies have reported that the numbers of cytotoxic NK cells are reduced in PF and PB of EN patients, accompanied by an overall decrease in NK cell activity, which suggests the functional impairment and diminished cytotoxicity of NK cells in EN (49, 50). However, this is inconsistent with our findings, and larger sample studies are needed to corroborate the results. γδ T cells represent only a small fraction of T lymphocytes (1-5%) (51). As primary subsets of γδ T cells, γδ2^+^ T cells and γδ1^+^ T cells perform distinct immunological roles. γδ1^+^ T cells are characterized by their regulatory and effector properties, while γδ2^+^ T cells are known for their cytotoxic actions, which are directed towards pathogen-specific features (52, 53). Research on γδ T cells in EN is sparsely documented. In our data, we found that the frequencies of γδ2^+^ T cells in γδ T cells were lower in the PB of EN, and the frequencies of PD-1^+^ cells in γδ1^+^ T and γδ2^+^ T cells were much higher compared to healthy controls. We speculate that γδ T cells might develop an exhausted phenotype following activation within the context of the prolonged chronic inflammation associated with EN. Intriguingly, Our results also demonstrated a correlation between the percentage of γδ1^+^ T cells in the peripheral blood of EN patients and both the severity of their disease and the presence of pain symptoms. Specifically, as the severity of the disease increased, the percentage of γδ1^+^ T cells also rose. Additionally, patients with pain symptoms had a higher percentage of γδ1^+^ T cells compared to those without symptoms. Conversely, the percentage of NKG2D^+^γδ2^+^ T cells decreased with increasing disease severity, and the percentage of γδ2^+^ T cells decreased in the presence of pain symptoms. These findings suggest that the imbalance in γδ1^+^ and γδ2^+^ T cell expression may be associated with disease progression and the presence of pain symptoms, which may lead to new diagnostic tools or treatment strategies for EN.

Furthermore, using machine learning techniques, we have pinpointed the key immune factors that are essential for constructing a diagnostic model capable of effectively differentiating between patients with EN and healthy individuals, including immature regulatory B cells, B10 cells, Th17 cells, homing memory Tc cells, MZ B cells, naive B cells, B cells, non-transformed B cells, transformed B cells, plasma cells, peripheral helper T cells, memory B cells, double-positive T cells, CD4^+^ T_CM_ cells, PD1^+^γδ2^+^ T cells, and classical transformed B cells. Interestingly, not all of these key factors identified by machine learning exhibited significant intergroup differences. This could suggest that the immunopathogenesis of EN is more intricate, possibly involving interactions among various immune subsets that have not yet been thoroughly investigated.

Compared to previous studies that focused narrowly on a select few individual immune markers (54–57), our study represents a significant advancement by utilizing multi-parameter flow cytometry to detect 70 immune indicators in the PB of patients with EN, comprehensively constructing a characteristic circulating immune profile in this patient population. However, this is still far from sufficient to fully elucidate the immune pathogenesis of EN and to enhance clinical diagnosis and treatment of this disease.

There are still some limitations in our study. Firstly, The majority of the EN patients enrolled in our study belonged to the combined subtype, with multiple lesions and forms. However, EN is a heterogeneous disease with various phenotypes, such as superficial peritoneal lesions, ovarian endometriomas, deep infiltrating endometriosis and Uterine Myometrium. We acknowledge the importance of considering the heterogeneity of EN, as it is a disease with various phenotypes and stages, which can influence immune responses. To address this shortcoming, more diverse and representative sample of EN subtypes will been included in future studies. Secondly, our result have shown the overlap in immune profiles between EN and UF patients, but whether UF will confuse these findings or obscure the specific immune changes associated with EN remains unknown. It would be valuable to investigate whether the immune dysfunctions observed are unique to EN or whether they are also present in other gynecological conditions, such as those represented by UF. This distinction is crucial when devising treatment strategies for EN, especially when considering immunotherapeutic interventions. Thirdly, through literature study (32, 55, 58), we found that most previous studies have similarly only used a limited number of immune indicators to explore the local immune environment, lacking comprehensive research on immune characteristics. Our study, which focused on peripheral blood immune profiles, did not include an analysis of the localized immune responses occurring within endometriotic lesions or peritoneal fluid. It is not well understood whether there are potential differences between systemic (peripheral) and localized immune responses in EN, and this gap in knowledge is a significant limitation of our research. We have emphasized the need for future research to study these local immune responses in detail and to compare the differences between peripheral and local immunity. Finally, acknowledging the intricate nature of immune system imbalances in disease development, we emphasize that the integration of a panel of immune markers, rather than reliance on individual markers alone, is crucial for a more holistic understanding of EN. To this end, the adoption of innovative technical approaches that extend beyond conventional multi-parameter flow cytometry is needed. The utilization of single-cell sequencing, mass cytometry, and sophisticated bioinformatics analysis techniques will be instrumental in unraveling the immune pathogenesis of EN from various perspectives and with greater depth (59, 60). We believe that these advanced methodologies will allow researchers to probe the immune landscape of EN with unprecedented resolution, identifying key cellular and molecular nodes within the disease’s immunopathogenesis. Taken together, further research to fortify the conclusions is necessary, including validation through a study with a larger sample size, longitudinal analyses to unravel the dynamics of local and systemic immune profiles before and after therapeutic interventions, and investigations into the clinical utility of the identified immune markers.

In conclusion, the present findings indicate that the peripheral immune disorder in EN is associated with B cells and their subsets, as well as Th and Tc cell subsets, mainly including Th2, Th17, Tc17, and Tfh cells, along with functional subsets of NK cells and γδ T cells. This study provides the first comprehensive depiction of the immune signature in PB of EN patients, although the detailed roles and underlying mechanism in EN require further investigation.

## Data Availability

The original contributions presented in the study are included in the article/**Supplementary Material**. Further inquiries can be directed to the corresponding authors.

## Supplementary material

Supplementary Figure 1 Comparison of age among the EN patients, UF-alone patients and healthy controls.

Supplementary Figure 2 The gates of unstained controls. **(A)** The gates of unstained controls for T cells, B cells, and NK cells; **(B)** The gates of unstained controls for distinct T subsets, including γδ T cells, CD4^+^ T cells, CD8^+^ T cells, and Treg cells; **(C)** The gates of unstained controls for NK subsets; **(D)** The gates of unstained controls for B subsets; **(E)** The gates of unstained controls for different stages of CD4^+^ and CD8^+^ T cells; **(F)** The gates of unstained controls for distinct subsets of CD4^+^ and CD8^+^ T cells. **(G)** The gates of unstained controls for different surface markers on γδ1^+^ and γδ2^+^ T cells.

Supplementary Figure 3 Unsupervised clustering analysis and principal component analysis (PCA) of assess the effectiveness of peripheral immune indexes in distinguishing the EN patients from the healthy controls. **(A)** Heat maps formed by unsupervised clustering analysis; **(B)** PCA of the percentages of total 70 peripheral immune indexes in the EN patients and the healthy controls. **(C)** Heat maps formed by unsupervised clustering analysis; **(D)** PCA of the percentages of the 25 peripheral immune indexes with significant differences between the EN patients and the healthy controls. PC1 refers to the first principal component, whereas PC2 indicates the second principal component.

Supplementary Figure 4 The models of distinguishing the EN patients from UF-alone patients. **(A)** The ROC curve of the efficiency of the diagnostic model constructed by one of random samplings using 70 immune indexes. **(B)** The AUC distribution of ROC curves from 1000 random samplings of the efficiency of the diagnostic model with 70 immune features. **(C)** The importance ranking calculated by the random forest model of the 70 immune cell subsets. **(D)** The average AUC distribution of ROC curves of the efficiencies of diagnostic models constructed by top important immune features. **(E)** The AUC distribution of ROC curves from 1000 random samplings of the efficiency of the diagnostic model with top 8 important immune features. **(F)** The importance ranking calculated by the random forest model for the top 8 important immune features.
