# Supplementary figures and images for: Peripheral immune characteristics and subset disorder in reproductive females with endometriosis
Source: Front Immunol. 2024 Nov 28;15:1431175. doi: 10.3389/fimmu.2024.1431175 (PMC11634862; doi:10.3389/fimmu.2024.1431175)

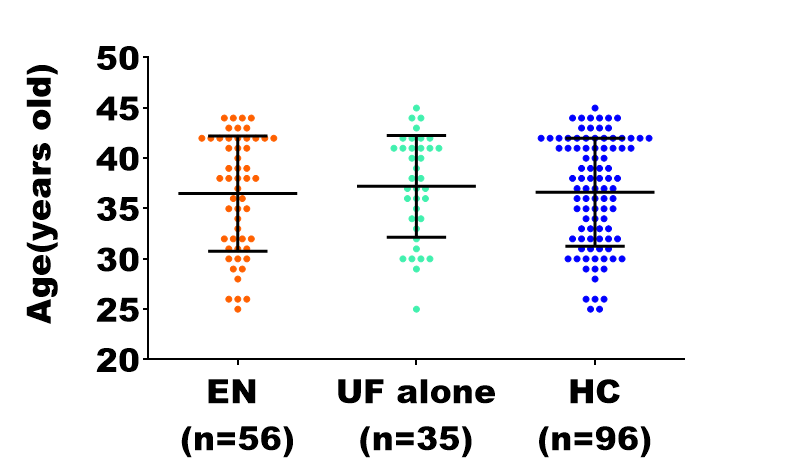

Supplement: Supplementary Figure 1 — Comparison of age among the EN patients, UF-alone patients and healthy controls. [file Image1.tif]

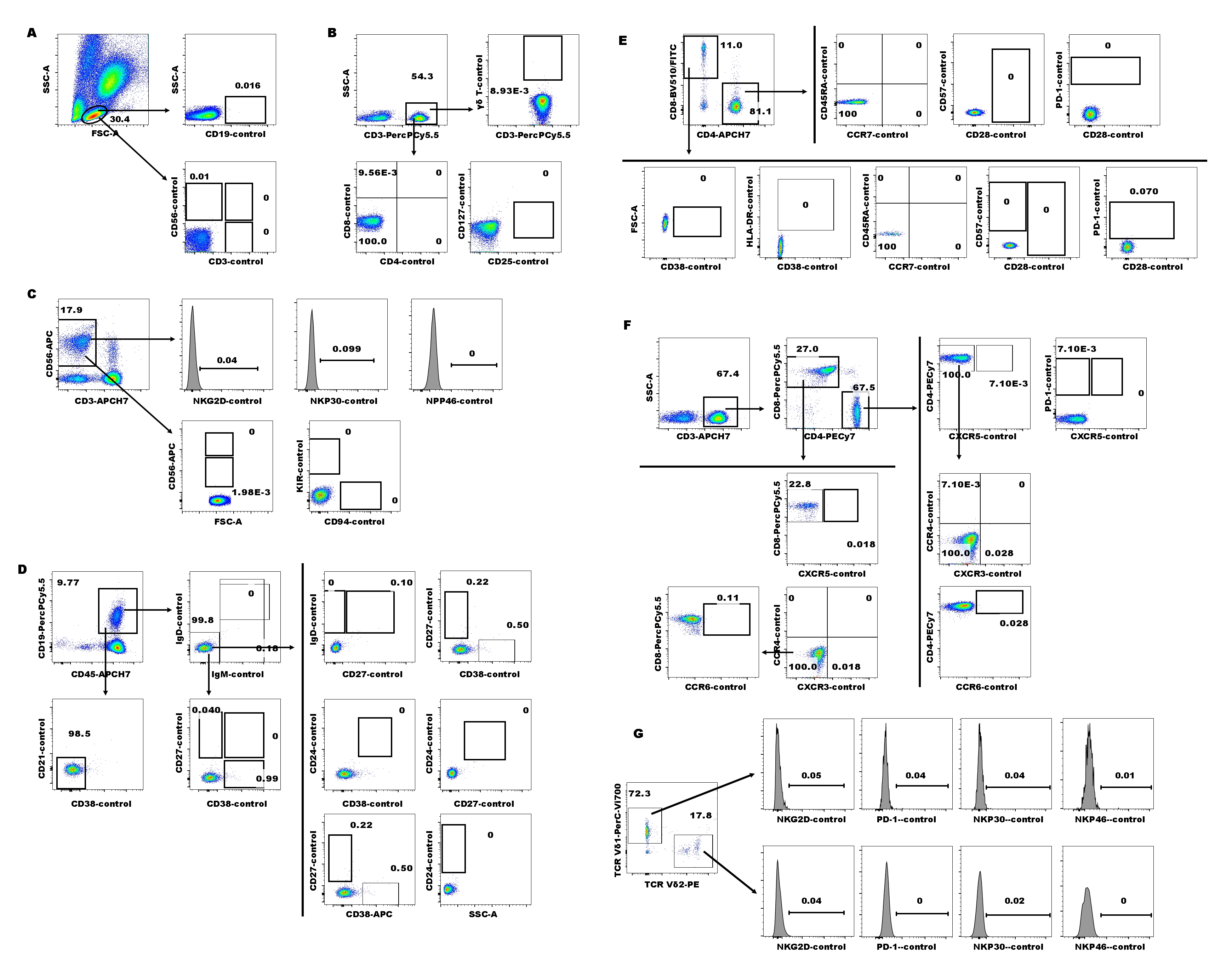

Supplement: Supplementary Figure 2 — The gates of unstained controls. (A) The gates of unstained controls for T cells, B cells, and NK cells; (B) The gates of unstained controls for distinct T subsets, including γδ T cells, CD4+ T cells, CD8+ T cells, and Treg cells; (C) The gates of unstained controls for NK subsets; (D) The gates of unstained controls for B subsets; (E) The gates of unstained controls for different stages of CD4+ and CD8+ T cells; (F) The gates of unstained controls for distinct subsets of CD4+ and CD8+ T cells. (G) The gates of unstained controls for different surface markers on γδ1+ and γδ2+ T cells. [file Image2.tif]

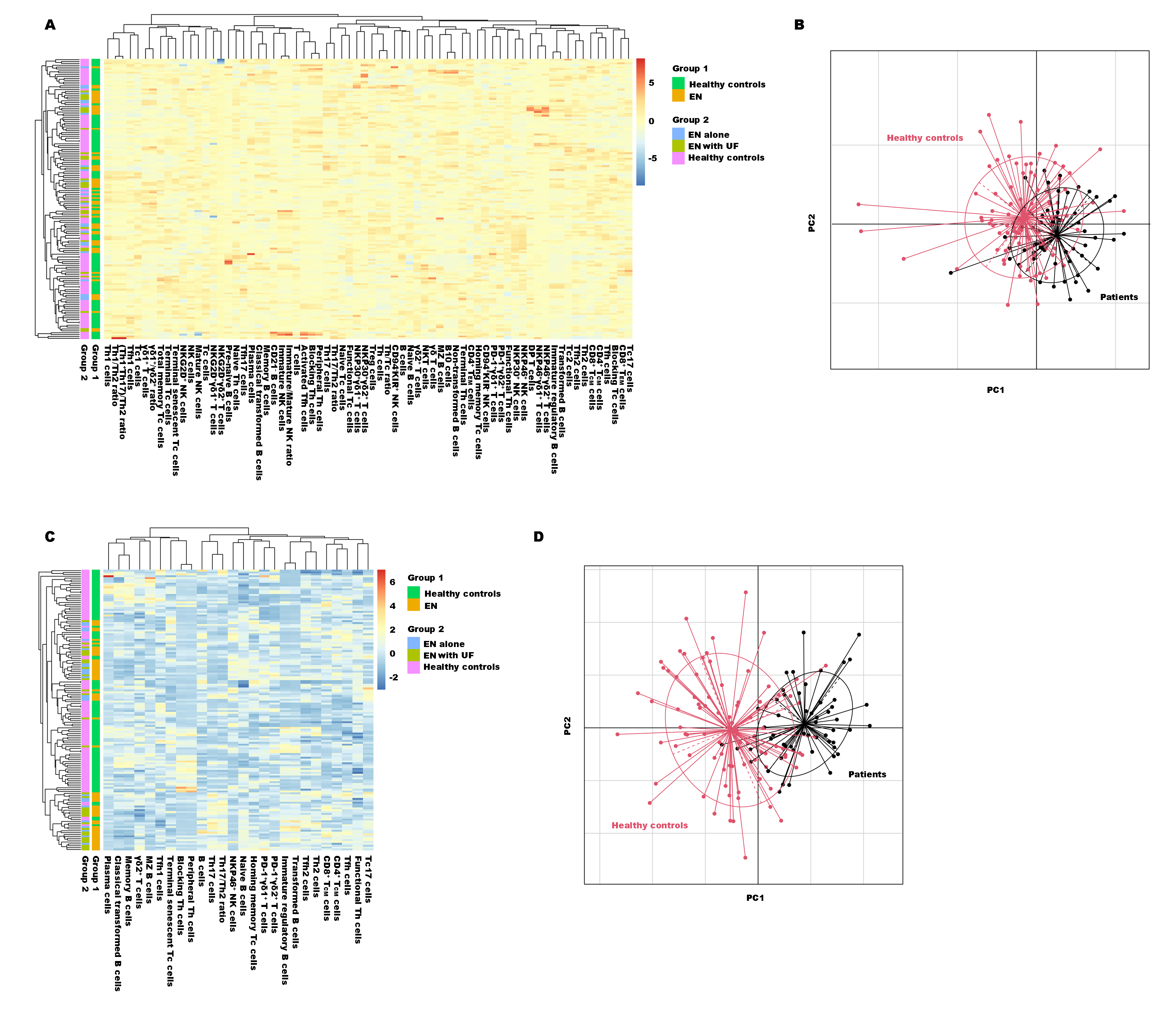

Supplement: Supplementary Figure 3 — Unsupervised clustering analysis and principal component analysis (PCA) of assess the effectiveness of peripheral immune indexes in distinguishing the EN patients from the healthy controls. (A) Heat maps formed by unsupervised clustering analysis; (B) PCA of the percentages of total 70 peripheral immune indexes in the EN patients and the healthy controls. (C) Heat maps formed by unsupervised clustering analysis; (D) PCA of the percentages of the 25 peripheral immune indexes with significant differences between the EN patients and the healthy controls. PC1 refers to the first principal component, whereas PC2 indicates the second principal component. [file Image3.tif]

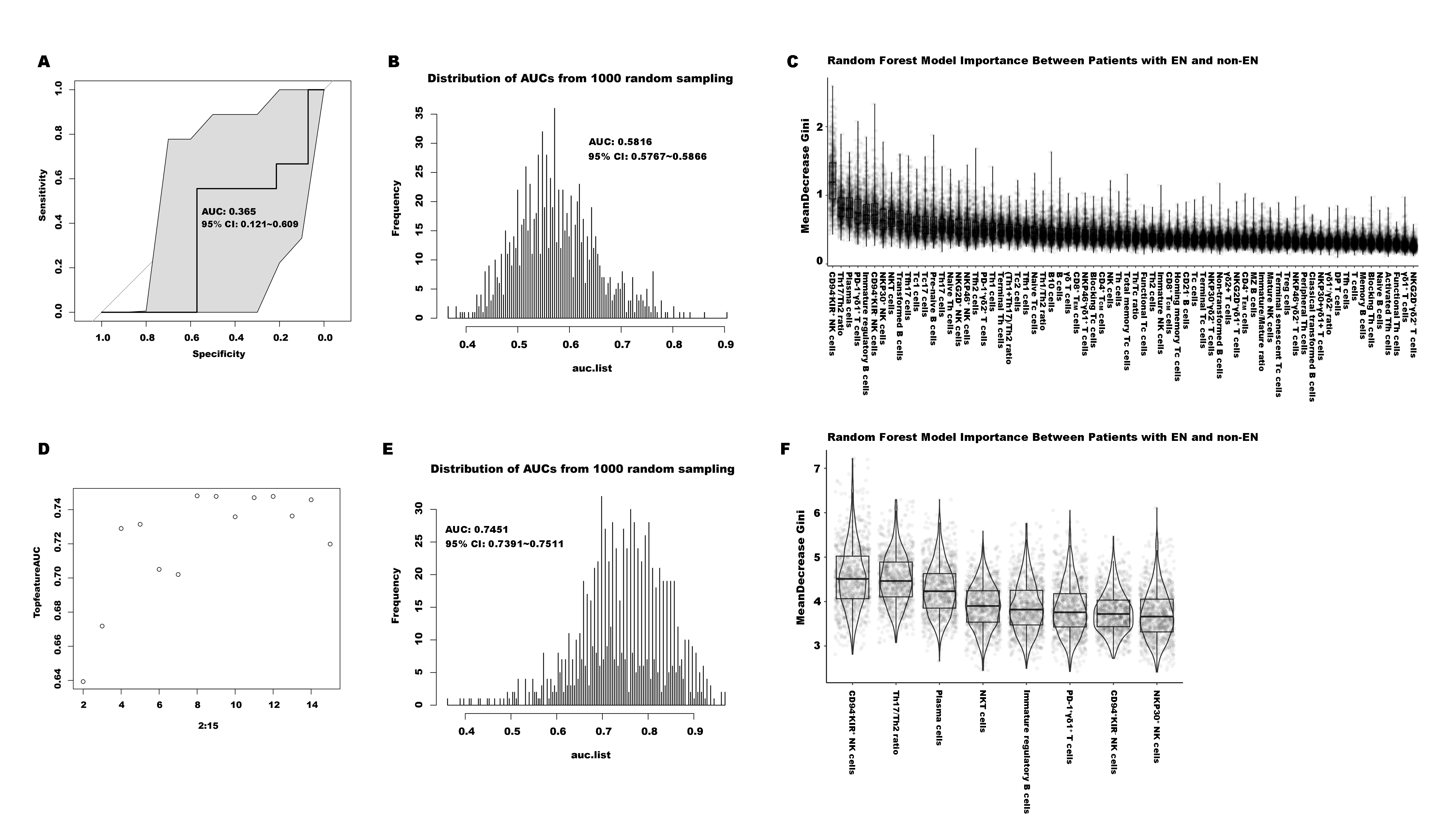

Supplement: Supplementary Figure 4 — The models of distinguishing the EN patients from UF-alone patients. (A) The ROC curve of the efficiency of the diagnostic model constructed by one of random samplings using 70 immune indexes. (B) The AUC distribution of ROC curves from 1000 random samplings of the efficiency of the diagnostic model with 70 immune features. (C) The importance ranking calculated by the random forest model of the 70 immune cell subsets. (D) The average AUC distribution of ROC curves of the efficiencies of diagnostic models constructed by top important immune features. (E) The AUC distribution of ROC curves from 1000 random samplings of the efficiency of the diagnostic model with top 8 important immune features. (F) The importance ranking calculated by the random forest model for the top 8 important immune features. [file Image4.tif]
